# Mapping Natural Dyes in Archeological Textiles by Imaging Mass Spectrometry

**DOI:** 10.1038/s41598-019-38706-4

**Published:** 2019-02-20

**Authors:** Annemarie Elisabeth Kramell, María García-Altares, Maria Pötsch, Ralph Kluge, Annekatrin Rother, Gerd Hause, Christian Hertweck, René Csuk

**Affiliations:** 1 Department of Organic Chemistry, Martin Luther University Halle-Wittenberg, 06120 Halle, Germany; 2 Department of Biomolecular Chemistry, Leibniz Institute for Natural Product Research and Infection Biology, Hans Knöll Institute, 07745 Jena, Germany; 3 Department of Electron Microscopy, Biocenter, Martin Luther University Halle-Wittenberg, 06120 Halle, Germany

## Abstract

Organic dyes of animal and plant origin have often been used by our ancestors to create textiles with polychromic ornamental patterns, and dyestuff analyses reveal how ancient cultures used these natural colorants. Mass spectrometry can characterize ancient colorants from these textiles, but its combination with separation techniques such as liquid chromatography requires the destruction of the pattern to extract organic dyes from the fabrics. In this study we applied mass spectrometry imaging (MS imaging) on colorful patterned textiles to show the spatial distribution of indigo-type and anthraquinone-type dyes. We evaluated different sample preparation techniques for matrix-assisted laser desorption/ionization time-of-flight (MALDI-TOF)-MS imaging, e.g. the production of imprints in TLC (thin layer chromatography) aluminum sheets and the embedding of the material in Technovit7100 to produce thin sections. Our protocol enabled the detection of indigo-type dyes directly on a historic textile of more than 2,000 years old embedded in Technovit7100. This is the first-time application of MALDI-TOF-MS imaging to map different organic dyestuffs on archeological remains.

## Introduction

Natural dyes have been used in almost all cultures, and almost all periods of time, in many everyday items including food, cosmetics and decoration. Their presence in archeological samples gives crucial information about the way of living during past times. For instance, many ancient cultures, such as ancient Peruvian cultures, used several types of natural organic dyes like indigo and carminic acid, to create highly complex ornamental patterns in their textiles, which reveals the knowledge of a wide range of skills to extract and to process natural dyes from plants and animals^1–3^.

To characterize natural colorants in historic textile samples, dyestuff analyses have been performed successfully on textile fragments or a few fibers using commonly (U)HPLC-DAD [(ultra) high performance liquid chromatography – diode array detection], (U)HPLC-ESI-MS/MS [(U)HPLC - electrospray ionization tandem mass spectrometry) or HPLC-APCI-MS/MS (HPLC - atmospheric pressure chemical ionization tandem mass spectrometry) combined with a previous extraction of textile materials^4–8^. In fact, MS is an excellent tool for the characterization of ancient colorants and the combination with different separation techniques allows a sensitive and selective determination of coloring components and degradation products. In this context, high separation efficiency is usually achieved by the application of LC^9^. In addition, the utilization of GC- (gas chromatography)^10,11^ and CE-MS (capillary electrophoresis)^12,13^ has been also described.

However, these analytical techniques require the destruction of the pattern to extract organic dyes from the fabrics, thus they are unable to provide information about the distribution of colorants in polychromic textiles with complex patterns.

On the other hand, MS imaging has evolved as a valuable technique to localize the distribution of organic molecules on solid samples, such as human or animal tissues, plant materials, and historic objects like paintings^14–17^. Compared with the large scale of publications dealing with MS imaging of peptides, proteins, lipids and carbohydrates, studies on imaging of colorants in complex samples are rather rare. However, a few examples are known. For instance, in the field of forensic science, MS Imaging was applied to investigate trace evidences like textile fibers, in which synthetic dyes were mapped in single nylon fibers and single acetate yarns using TOF-SIMS (time-of-flight secondary ion mass spectrometry)^18^ or IR-MALDESI (infrared matrix-assisted laser desorption electrospray ionization)^19^.

(MA)LDI-TOF-MS is a softer ionization method compared with TOF-SIMS^14,15^ and therefore well-known as reliable method for the identification of natural dyes and pigments in complex multicomponent solid samples of archaeological interest^20–24^, and was successfully applied for the direct analysis of natural dyes on the surface of single textile fibers^25^. However, MALDI-TOF-MS imaging experiments on textile fabrics are not yet described in the literature.

Direct mass spectrometric methods such as DART- (direct analysis in real time) and flowprobe-ESI-MS are also known for analyses of natural dyes on historic textiles^26–28^. Nevertheless, the spatial resolution of these techniques (from 630 µm to mm range^29–31^) is not sufficient to visualize the distribution of dyestuffs in polychromic textiles with yarns, which often have diameters smaller than 1 mm. In comparison, most commercial MALDI-TOF-MS instruments routinely achieve lateral resolution of 20 µm and special modifications of the imaging source combined with an appropriate sample preparation and data processing even enable lateral resolutions <10 µm^32^.

Here we report the optimization of sample preparation techniques, usually applied in the investigation of animal and plant tissues, to study archeological remains by MALDI-TOF-MS imaging. In this context, the challenge was to handle topography effects of textile fabrics. Moreover, the first-time application of MALDI-TOF-MS imaging on polychromic recent and historic textiles dyed with indigo-type and anthraquinone-type dyes was presented.

## Material and Methods

### Chemicals and materials

Indigo and alizarin (97%) were purchased from ACROS Organics; indirubin (≥98%), 9-aminoacridine (9-AA, ≥99.5%), erythrosin B (≥95%), 2-iodobenzoic acid (≥99%) and Universal MALDI matrix [1:1 mixture of 2,5-dihydroxybenzoic acid (DHB) and α-cyano-4-hydroxy-cinnamic acid (α-CCA)] were obtained from Sigma-Aldrich, carminic acid (≥96%) from Fluka Analytical, lucidin from Cfm Oskar Tropitzsch GmbH (Marktredwitz), tetrahydrofuran (HPLC grade) from Roth, formic acid (99–100%), trifluoroacetic acid, ethanol, methanol and acetonitrile (all solvents HPLC gradient grade) from VWR, and TLC (thin-layer chromatography) plates (ALUGRAM Sil G) were purchased from Macherey-Nagel. Purpurin has been obtained from Aldrich’s collection of rare chemicals and rubiadin has been synthesized according to procedures of Takano *et al*.^33^. Double distilled water was produced in a distillation apparatus bought from Gebr. Rettberg GmbH. An extended tabby weave with blue warps (wool fibers previously vat dyed using natural indigo) and red wefts (wool fibers previously mordant dyed using alum and cochineal) was handcrafted by a textile archaeologist. Recent animal- and plant-derived fibers dyed with synthetic indigo or E120, a food dye extract from scale insects (*Dactylopius coccus* Costa), were used for first tests (dyeing processes were performed as previously reported^28,34^).

### Historical textile sample

Samples consisted of wool or cotton and were either taken from a shirt (object ID: 95MNIM5–43) excavated from the archaeological site of Niya (probable period of use: 2^nd^ century BC – 5^th^ century AD^35^) found in the southern edge of the Tarim Basin (Xinjiang Uyghur Autonomous Region, China) or originate from a fabric fragment (object ID: VA5704) assigned to the Ichma culture (1100–1440 AD, ancient Peruvian culture).

### Instruments

For topographic documentations a CX41- (transmission light microscope, from Olympus) and a VHX-700FD optical microscope (digital microscope from Keyence) were used.

MALDI-TOF-MS data were acquired using an ultrafleXtreme spectrometer from Bruker Daltonics (MS scan range: 0–2,100 *m/z* or 0–1,500 *m/z*, Pulsed Ion Extraction: 70 ns, reflector positive and negative mode) equipped with an ultraviolet laser (smartbeam-II laser of 1,000 Hz, laser power: 20–50%). Desorption and ionization was performed with the assistance of a matrix. For the analysis of anthraquinones, 9-AA was dissolved in ethanol:water (7:3 *v/v*) with trace added amounts of erythrosin B and 2-iodobenzoic acid for internal calibration of the spectra; for indigoid dyes, Universal MALDI matrix was dissolved in acetonitrile (7 g/L for Imaging experiments) or acetonitrile:water (3:7 *v/v*) containing 0.1% (*v/v*) trifluoroacetic acid (for investigation of single fibers). Calibration of the acquisition method in reflector positive was performed externally using Peptide Calibration Standard II (Bruker Daltonics) containing Bradykinin1–7, Angiotensin II, Angiotensin I, Substance P, Bombesin, ACTH clip1–17, ACTH clip18–39, and Somatostatin 28. Spectra were calibrated internally using [M − H]^−^ of erythrosin B, 2-iodobenzoic acid and 9-AA in reflector negative mode and [M + H]^+^ and [2 M + H]^+^ of α-CCA and DHB in reflector positive mode.

For method optimization, authentic standard compounds were suspended or dissolved in methanol, mixed with a solution of the matrix compound and spotted onto the surface of a MALDI target plate with subsequent evaporation of the solvent. Single fibers or bunches of fibers were fixed at their two ends onto ITO (indium-tin-oxide) coated glass slides from Bruker Daltonics with conductive tape, and measurements were carried out in the middle part of fibers positioned in close contact with the conductive slides. For studies on fibers, imprints or fabric sections the matrix was applied automatically with an ImagePrep device of Bruker Daltonics. For method optimization, different solvents and solvent ratios were tested. Image acquisitions of Technovit7100-embedded fabrics and imprints were performed at a spatial resolution of 75 µm to 100 µm and spectral data were processed using flexAnalysis as well as flexImaging software from Bruker Daltonics. For advanced image visualization of MALDI-TOF-MS imaging data, SCiLS Lab 2015 software from SCiLS GmbH was used (using the settings: no normalization and weak denoising).

### Production of Technovit7100-embedded fabric sections

Samples were transferred into Technovit7100 (Heraeus Kulzer, Wehrheim, Germany) infiltration solution at room temperature and infiltrated 4 times in vacuum to remove air bubbles. The infiltration solution was changed 5 times over two days. After transfer to 4 °C, the infiltration solution was changed three times (each step at least 4 hours). Finally, the samples were polymerized in Technovit7100 embedding solution at 4 °C for 12 hours.

Semithin sections (3 µm) were made with an OmU3 ultramicrotome (Reichert, Vienna, Austria), placed onto a droplet of water at ITO coated glass slides and dried for 12 hours at 45 °C. Finally, sections were coated with 9-AA or Universal MALDI matrix.

### Production of Imprints (modified according to Cabral *et al*.)

TLC plates were wetted with acetonitrile:water (1:1 *v/v*) containing 0.1% formic acid and 1% tetrahydrofuran, and a textile fragment was placed between two TLC plates. This assembly was pressed for 10 s using a hot metal plate (190 °C). Finally the TLC plates were air-dried, fixed onto ITO coated glass slides with conductive tape and 9-AA or Universal MALDI matrix was applied^36^.

## Results and Discussion

### Optimization of ionization yields with authentic dye references

**Figure 1:**
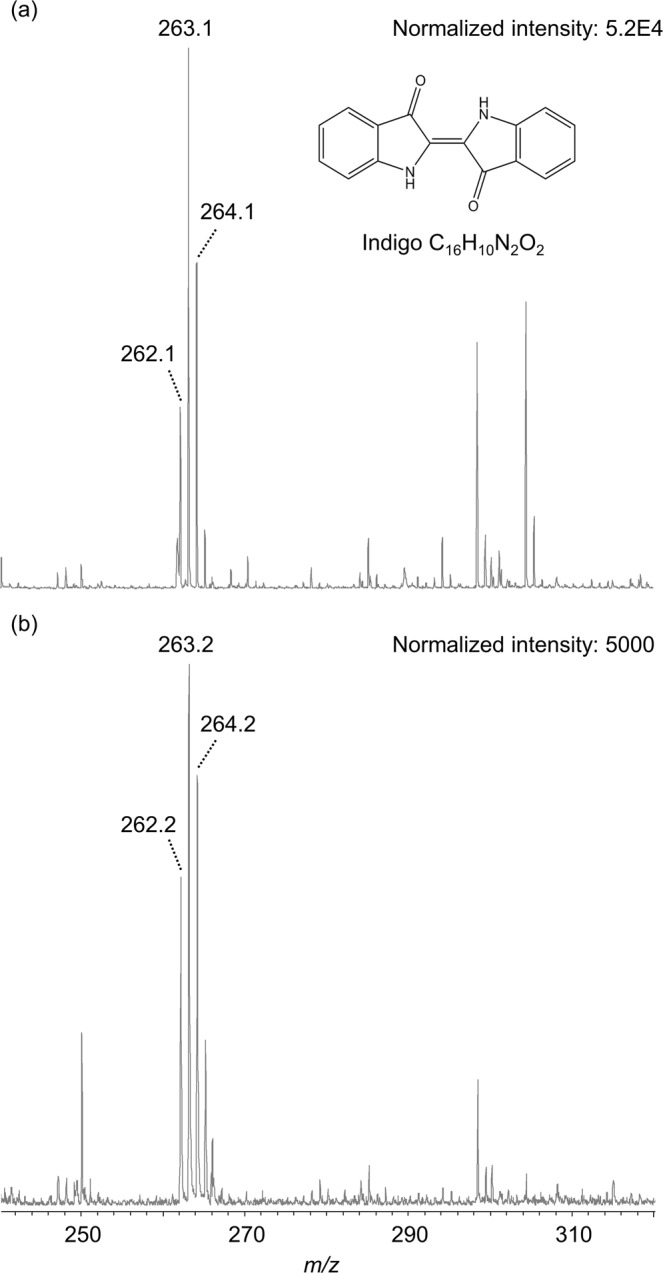
MALDI-TOF-MS spectra of (**a**) an authentic indigo reference and (**b**) blue-colored historic fibers (sample ID VA57042). Measurements were carried out with the assistance of Universal MALDI matrix in reflector positive mode. Indigo-type dyestuffs were detected as radical cation [M]^•+^ (*m/z* 262) and protonated ion [M + H]^+^ (*m/z* 263). Note: Besides these signals, a mass peak at *m/z* 264 was detected. A similar signal was also observed in positive ion MALDI-TOF-MS and TOF-SIMS spectra of indigo by Xu *et al*.^41^ and Lee *et al*.^42^. Presumably, it is a product of a redox reaction ([M + 2H]^•+^ as putative species, compare^43,44^).

Initially, we investigated the ionization yield of genuine standard compounds (anthraquinone- and indigo-type dyes such as carminic acid, alizarin, lucidin, purpurin, rubiadin, indigo and indirubin) with and without assistance of a matrix. The application of matrices led to increased analyte signal intensities, and best results were achieved for indigo-type dyestuffs with Universal MALDI matrix in positive ionization mode and for anthraquinone colorants with 9-AA in negative ionization mode. Anthraquinones were detected as radical anions [M]^•−^ and deprotonated ions [M − H]^−^ (Supplementary Figs S1 and S2). For the isomers indigo (Fig. 1a) and indirubin, radical cations [M]^•+^ at *m/z* 262.1 and protonated ions [M + H]^+^ at *m/z* 263.1 were observed. Thus, this method is suitable to characterize anthraquinone- and indigo-type dyes, the most important “ancient” natural organic dyestuffs to create blue and red hues.

### MALDI-TOF-MS imaging experiments on textile fibers

In the next step, we attempted the detection of organic dyestuffs by MALDI-TOF-MS imaging directly from single or bunches of fibers previously dyed using indigo, and using alum and cochineal extracts with carminic acid being the main colorant. We could ionize and detect the analytes from these reference fibers. Moreover, we could also detect indigo-type dyes on a few blue cotton fibers from an ancient Peruvian textile (sample ID: VA57042). Here, mass peaks at *m/z* 262.2 and 263.2 were detected with assistance of Universal MALDI matrix in positive mode (Fig. 1b) and indicate the utilization of the vat dye indigo as blue colorant. This archeological sample had been previously investigated by LC-MS/MS and HPLC-DAD experiments that confirmed the presence of the indigo-type dyes indigo and indirubin^34^.

However, the direct analysis of fiber materials by MALDI-TOF-MS imaging proved to be suboptimal in some cases. First, the fibers have to be placed in close contact with a conductive probe holder, and differences in the z-axis position have to be avoided or corrected (for example with a calibrant and post-run recalibration) because differences in ionization position may impel differences in signal intensities or shifts in the flight-time^25,37^. Furthermore direct investigations on sensitive fiber materials, e. g. silk, may cause damage to fibers by laser irradiation, impeding the analysis of the sample.

Since the direct investigation of textiles by MALDI-TOF-MS imaging can lead to irregular signal intensity, mass shifts, and sample damage, we aimed at adapting different sample preparation techniques for MS imaging. In this context, we adopted the technique of immersion of tissues in paraffin to produce thin sections, well-known from histological investigations on animal and human tissues and organs^38,39^. Moreover we produced imprints of the textile sample in TLC aluminum sheets. This approach has been described to investigate the surface of plant tissues and fruits by DESI (desorption electrospray ionization)-MS imaging^36^.

### MALDI-TOF-MS imaging experiments on a reference textile fabric

**Figure 2:**
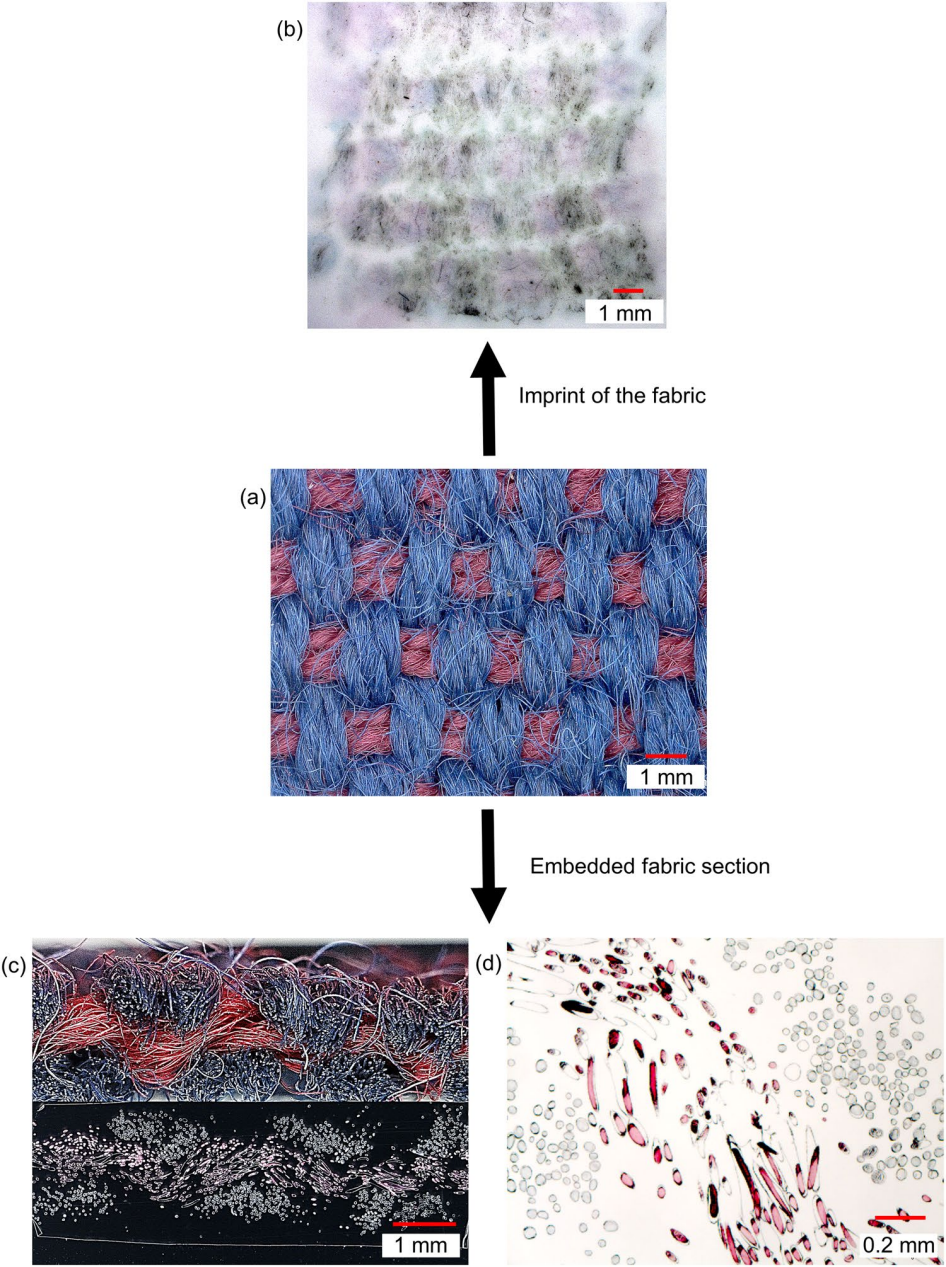
(**a**) Extended tabby weave with blue warps and red wefts. (**b**) Optical image of an imprint of the extended tabby weave onto the surface of a TLC plate. (**c**) A cut along the red weft and an optical image of a Technovit7100-embedded fabric section. (**d**) Magnification of a fabric section with blue and red areas (bright-field microscopy).

We tested these two different sample preparation methods, imprinting and embedding, using a recently woven extended tabby weave with blue warps and red wefts (Fig. 2a). This textile was manufactured by a textile archaeologist applying traditional weaving and coloring techniques. The fabric consists of blue yarns colored with the vat dye indigo and red yarns dyed with cochineal (carminic acid being the main colorant).

For imprinting, dyestuffs of this colored textile were transferred to TLC aluminum sheets with the assistance of solvent extraction and heating during blotting. For embedding, a piece of this textile was embedded in Technovit7100, cut (3 or 5 µm slices), and the sections were mounted on ITO coated glass slides. For each sample preparation, we produced two replicated samples. One imprint/section was coated with Universal MALDI matrix and analyzed by MALDI-TOF-MS imaging reflector positive mode, while the other replicated imprint/section was coated with 9-AA matrix and analyzed in reflector negative mode. A resulting imprint and a fabric section of the examined extended tabby weave are presented in Fig. 2b–d.

**Figure 3:**
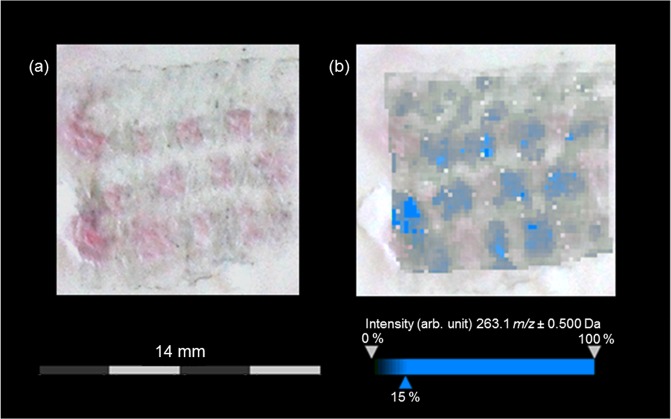
MALDI- TOF-MS imaging results for an imprint of the extended tabby weave with blue warps and red wefts dyed with indigo or cochineal. (**a**) Optical image of the imprint of the extended tabby weave onto the surface of a TLC plate. (**b**) Visualization of indigo (*m/z* 263.1 [M + H]^+^) measured in reflector positive mode with Universal MALDI matrix (half-transparent overlay of the optical image and MALDI-TOF-MS imaging data).

Producing imprints is fast and simple compared to embedding and cutting. In addition, indirect MS imaging analyses of imprints enable a reuse of sample materials for more investigations. Because of their non-destructive character indirect analyses are suited especially for investigating archaeological samples being mostly available only in tiny amounts. The optical image of an investigated imprint showed the characteristic pattern of the extended tabby weave with bluish and reddish areas (Fig. 3). The visualization of bluish regions by MALDI-TOF-MS imaging is displayed in Fig. 3 (detected dye: indigo with *m/z* 263 [M + H]^+^). However, the imprinting technique was - under the selected measurement conditions – of limited use for mapping the low content of red colorants onto the surface of TLC plates. Furthermore, the colored regions of the transferred weaving pattern are blurry because of diffusion processes of the colorants. Thus, this sample preparation is prone to analyte delocalization.

**Figure 4:**
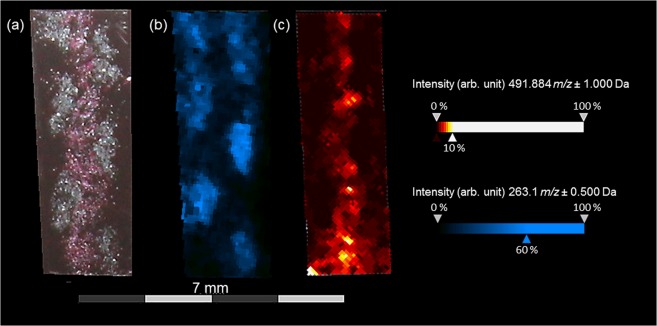
MALDI-TOF-MS imaging results for Technovit7100-embedded fabric sections of the extended tabby weave with blue warps (dyed with indigo) and red wefts (dyed with cochineal). (**a**) Optical image of a Technovit7100-embedded fabric section. (**b**) Visualization of indigo (*m/z* 263.1 [M + H]^+^) measured in reflector positive mode with Universal MALDI matrix. (**c**) Visualization of carminic acid (*m/z* 491.1 [M − H]^−^ and 492.1 [M]^•−^) measured in reflector negative with 9-AA matrix.

In comparison, the distribution of colored fibers within the textile was fixed through the embedding process (Fig. 2d). Related MALDI-TOF-MS images of Technovit7100 -embedded fabric sections (Fig. 4) display the distribution of carminic acid (*m/z* 491.1 [M − H]^−^) and indigo (*m/z* 263.1. [M + H]^+^). The spatial distribution of the detected dyestuffs reflected the course of the threads. Since direct imaging analyses on Technovit7100-embedded fabric sections were more suitable than indirect analyses of imprints for mapping red and blue dyestuffs of the examined fabric, we used the embedding sample preparation for further investigations on a historic textile.

### Case study: MALDI-TOF-MS imaging experiments on a historical textile fabric

**Figure 5:**
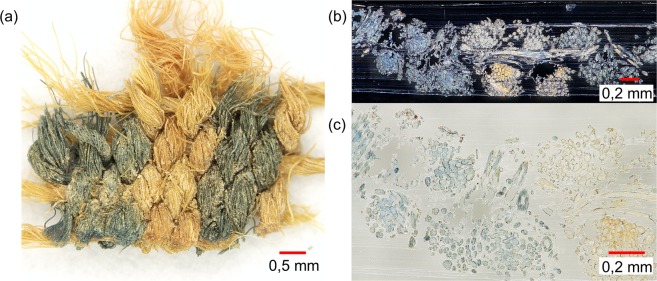
(**a**) Historic fabric fragment (sample ID: 95MNIM5-43B) with blue yarns and uncolored yarns. (**b**) Optical image of a Technovit7100-embedded fabric section. (**c**) Magnification of a fabric section with blue and undyed areas.

**Figure 6:**
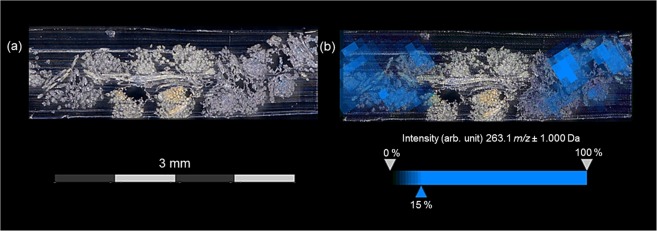
MALDI-TOF-MS imaging results for Technovit7100-embedded fabric sections of a historic fabric fragment (sample ID: 95MNIM5-43B). (**a**) Optical image of a Technovit7100-embedded fabric section. (**b**) Visualization of indigo (*m/z* 262. 1 [M]^•+^ and *m/z* 263.1 [M + H]^+^) and measured in reflector positive mode with Universal MALDI matrix (half-transparent overlay of an optical image and MALDI-TOF-MS imaging data).

MALDI-TOF-MS imaging experiments were performed on Technovit7100-embedded fabric sections of a historic fabric fragment from the archaeological finding site of Niya, China (Fig. 5). This approximately 2,000 years old woolen fragment (sample ID: 95MNIM5-43B) belongs to a decorative element of a shirt and consists of blue and uncolored twisted yarns arranged in a striped pattern^40^. Mass peaks at *m/z* 262.1 and 263.1 were detected with assistance of Universal MALDI matrix in positive mode on the blue regions and reveal the use of the blue colorant indigo for textile dyeing. A related MALDI-TOF-MS image (Fig. 6) correlates the blue regions in the optical image and the distribution of indigo in the MS image.

## Conclusions

In this paper, we reported on the application of MALDI-TOF-MS imaging to study archeological textiles respecting their colorful pattern. The optimization of the experimental setup was performed on multicolored reference materials and historic fibers dyed with indigo-type and anthraquinone dyestuffs - the most important representatives of natural organic dyestuffs for the generation of blue and red hues. For imaging experiments on non-flat fabric surfaces, we adopted two different sample preparation methods that are typically applied for MS imaging analysis on animal and human tissues and plant materials. First, textiles were imprinted onto the surface of aluminum TLC sheets by solvent extraction blotting with thermal assistance. This indirect sampling technique produced imprints of the blue-red fabric structure and enables mapping of the blue colorant indigo from the surface of matrix coated TLC plates. However, this preparation leads to analyte delocalization. The second sample preparation method comprised dyestuff imaging directly from the surface of thin matrix-coated Technovit7100-embedded fabric sections. With this approach we could detect blue and red dyestuffs directly on the fixed multicolored fibers, and we show that their spatial visualization matches the characteristic pattern of the examined fabric section.

MALDI-TOF-MS imaging is a promising approach for mapping different natural products such as organic dyestuffs on objects of archaeological interest. It generates a molecular picture on complex archaeological samples with lateral resolution in the µm range that enables the visualization of subtleties of an object, e. g. the course of yarns or fibers in textile patterns. The opportunity to visualize the distribution of different kinds of molecules with a single analytical method offers tremendous resources for archaeological research, since the coloration of historical objects usually consists of a mixture of various chromophore substances, and the knowledge about the combination and the utilization of these components provides an insight into the technology of a long gone era.

## Supplementary information

Supplementary information Mapping Natural Product Dyes in Archeological Textiles by Imaging Mass Spectrometry

## Data Availability

The data generated during and/or analyzed during the current study are available from the corresponding author on reasonable request.
