# Supplementary material for: Mapping Natural Dyes in Archeological Textiles by Imaging Mass Spectrometry
Source: Sci Rep. 2019 Feb 20;9:2331. doi: 10.1038/s41598-019-38706-4 (PMC6382771; doi:10.1038/s41598-019-38706-4)
Supplement: Supplementary file 1 — Supplementary information Mapping Natural Product Dyes in Archeological Textiles by Imaging Mass Spectrometry [file 41598_2019_38706_MOESM1_ESM.pdf]

Supplementary information

Mapping Natural Dyes in Archeological Textiles by Imaging Mass Spectrometry

Annemarie Elisabeth Kramell[1,*], María García-Altares[2], Maria Pötsch[2], Ralph Kluge[1], Annekatrin Rother[3], Gerd Hause[3], Christian Hertweck[2] & René Csuk[1]

[1]Department of Organic Chemistry, Martin-Luther-University Halle-Wittenberg, 06120 Halle, Germany

[2]Department of Biomolecular Chemistry, Leibniz Institute for Natural Product Research and Infection Biology, Hans Knöll Institute, 07745 Jena, Germany

[3]Department of Electron Microscopy, Biocenter, Martin-Luther-University Halle-Wittenberg, Germany

*Corresponding Author: annemarie.kramell@chemie.uni-halle.de

Table of contents

Figure S1. MALDI-TOF-MS spectra of alizarin, rubiadin and purpurin …………………………………...S-2

Figure S2. MALDI-TOF-MS spectra of lucidin and carminic acid ……………………...………...…..……S-3

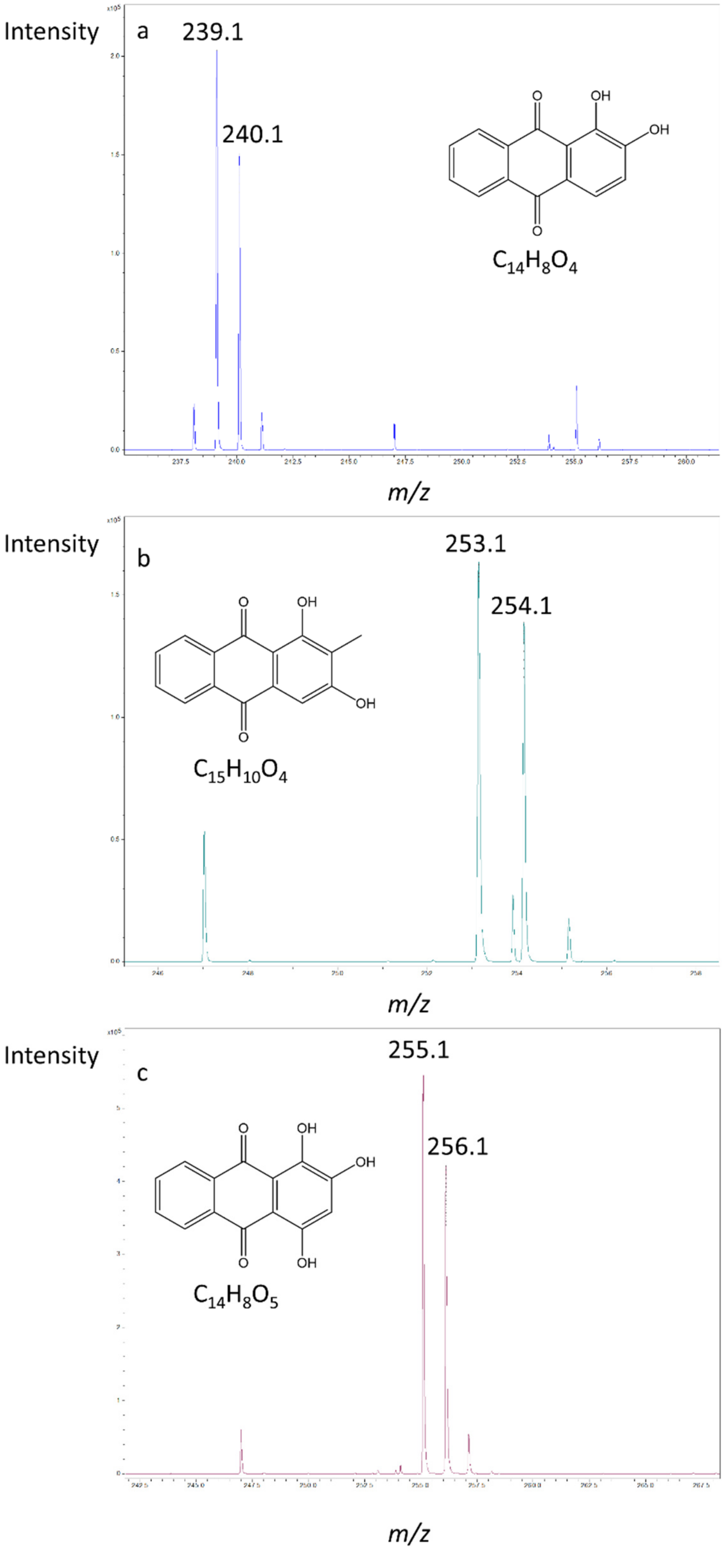

Figure S1. MALDI-TOF-MS spectra of (a) alizarin, (b) rubiadin and (c) purpurin. Measurements were carried out with the assistance of 9-AA in reflector negative mode. Anthraquinones detected as radical anions $[M]^{\bullet-}$ and deprotonated ions $[M\text{-}H]^-$.

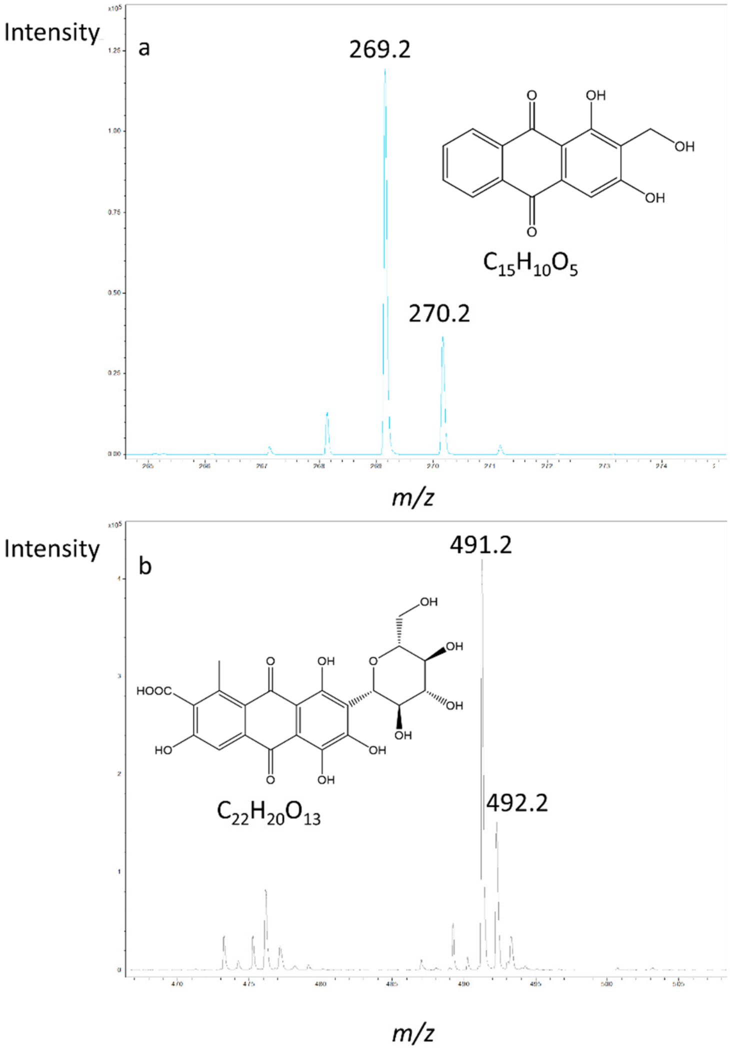

Figure S2. MALDI-TOF-MS spectra of (a) lucidin and (b) carminic acid. Measurements were carried out with the assistance of 9-AA in reflector negative mode. Anthraquinones detected as radical anions $[M]^{\bullet-}$ and deprotonated ions $[M\text{-}H]^{-}$.
